# Genome-Wide Association Study of Lint Percentage in *Gossypium hirsutum* L. Races

**DOI:** 10.3390/ijms241210404

**Published:** 2023-06-20

**Authors:** Yuanyuan Wang, Xinlei Guo, Xiaoyan Cai, Yanchao Xu, Runrun Sun, Muhammad Jawad Umer, Kunbo Wang, Tengfei Qin, Yuqing Hou, Yuhong Wang, Pan Zhang, Zihan Wang, Fang Liu, Qinglian Wang, Zhongli Zhou

**Affiliations:** 1 Collaborative Innovation Center of Modern Biological Breeding of Henan Province, Henan Institute of Science and Technology, Xinxiang 453003, China; 2 State Key Laboratory of Cotton Biology, Institute of Cotton Research, Chinese Academy of Agricultural Sciences, Anyang 455000, Chinaliufcri@163.com (F.L.); 3 Hainan Yazhou Bay Seed Laboratory, National Nanfan Research Institute of Chinese Academy of Agriculture Sciences, Sanya 572025, China; 4 Institute of Crop Science, Chinese Academy of Agricultural Sciences, Beijing 100081, China; 5 School of Agricultural Sciences, Zhengzhou University, Zhengzhou 450001, China

**Keywords:** *G. hirsutum* race, semi-wild cotton, lint percentage, genome-wide association study

## Abstract

Lint percentage is one of the most essential yield components and an important economic index for cotton planting. Improving lint percentage is an effective way to achieve high-yield in cotton breeding worldwide, especially upland cotton (*Gossypium hirsutum* L.). However, the genetic basis controlling lint percentage has not yet been systematically understood. Here, we performed a genome-wide association mapping for lint percentage using a natural population consisting of 189 *G. hirsutum* accessions (188 accessions of *G. hirsutum* races and one cultivar TM-1). The results showed that 274 single-nucleotide polymorphisms (SNPs) significantly associated with lint percentage were detected, and they were distributed on 24 chromosomes. Forty-five SNPs were detected at least by two models or at least in two environments, and their 5 Mb up- and downstream regions included 584 makers related to lint percentage identified in previous studies. In total, 11 out of 45 SNPs were detected at least in two environments, and their 550 Kb up- and downstream region contained 335 genes. Through RNA sequencing, gene annotation, qRT-PCR, protein–protein interaction analysis, the *cis*-elements of the promotor region, and related miRNA prediction, *Gh_D12G0934* and *Gh_A08G0526* were selected as key candidate genes for fiber initiation and elongation, respectively. These excavated SNPs and candidate genes could supplement marker and gene information for deciphering the genetic basis of lint percentage and facilitate high-yield breeding programs of *G. hirsutum* ultimately.

## 1. Introduction

Cotton is one of the most important cash crops, which supplies raw material to more than 50 industries globally and is extensively planted in more than 70 countries throughout the world [1]. Of the four cultivated species, upland cotton (*Gossypium hirsutum* L.), a main supplier for the textile industry, contributes approximately 95% of cotton production, owing to its high yield, attractive fiber quality, and wide adaptability [2,3]. Developing high fiber yield has always been one of the essential and constant targets in cotton breeding and cultivation, for yield is the foundation for the benefit of cotton planting [4]. Lint percentage (LP) is the fraction of fiber weight in seed–cotton weight, which is one of the most essential yield components and an important economic index for cotton planting [5,6,7]. It is close to cotton fiber yield and is significantly positively related to cotton yield but negatively correlated with the seed index. However, its genetic architecture is not fully understood; discovering the genetic variation and the candidate genes is still greatly meaningful for underlying lint percentage.

Lint percentage is a complex quantitative trait controlled by quantitative trait loci (QTL), which is also significantly influenced by different natural environments [8]. Moreover, the complex correlation between lint percentage and other fiber traits makes it more difficult to improve yield and quality traits simultaneously [3], only depending on traditional breeding techniques. Detecting QTL-linked or QTL-associated markers and applying for marker-assisted selection (MAS) breeding are effective for precluding the disturbance of environments, effectively breaking the negative correlation among traits, improving the efficiency of breeding. Linkage mapping is a traditional method for QTL/gene detection. The first cotton linkage map reported 11 linked groups located on chromosomes and mapped some QTL, including two for LP [9]. Since then, linkage mapping has been widely utilized in cotton with the construction of high-density molecular genetic maps using the populations from limited parents. Ample QTL for lint percentage were yielded using interspecific populations [10] and intraspecific populations [7,10,11,12,13]. These QTL provided meaningful insight for deciphering the genetic basis for lint percentage; however, the time consumption of constructing a mapping population and the low mapping accuracy of linkage analysis limited the exploitation of QTL through MAS breeding [14,15]. On the contrary, association mapping, as an alternative strategy, has lots of advantages, such as high resolution, no need for population construction, great efficiency, and low cost [5]. In recent years, association mapping also has been widely used for excavating marker–trait associations, which helps dissect the genetic basis of various complex traits in cotton [16,17,18,19]. Through genome-wide association mapping, a large number of SNPs associated with lint percentage have been discovered [20]. These associated markers supplied abundant marker resources and will be helpful for lint percentage improvement of modern cotton cultivars.

However, upland cotton has been experiencing domestication and breeding for thousands of years. The repeated selection of closely related genotypes has dramatically reduced the genetic basis of modern cotton cultivars, which has greatly hindered the breeding progress of yield breeding in *G. hirsutum* [21,22]. *G. hirsutum* races, also named semiwild cotton, are the progenitors of cultivated *G. hirsutum*, which have much more abundant phenotypic and genetic variations compared to the cultivars. Furthermore, no reproductive isolation between wild and cultivated types promoted the genetic mechanism analysis of complex traits. In the past years, with *G. hirsutum* races, several researchers executed linkage mapping, committing to broadening the genetic base for improving yield [23,24,25,26], fiber quality [22,27], and fiber color [25,28]. In our previous reports, SSR and SNP markers were selected to implement genome-wide association mapping for abiotic stress resistance, including glyphosate resistance [29] and drought resistance [30]. Furthermore, we performed a transcriptome analysis of *G. hirsutum* race accessions under alkali-salt stress and excavated the related candidate genes and signaling pathways [31]. The results above provided prolific information on markers and gene resources for the genetic improvement of cultivated upland cotton.

To detect the genetic architecture associated with lint percentage and the related candidate genes, we used the Cotton80KSNP chip to genotype the association mapping panel, comprising 188 accessions of *G. hirsutum* races and one cultivar (TM-1); we also investigated the lint percentage from five different environments and conducted the genome- wide association study (GWAS) to detect the genetic loci associated with lint percentage. Finally, we predicted the candidate genes that might affect lint percentage based on gene annotation and the gene expression pattern during fiber development. The results not only provided molecular markers and candidate genes for further understanding the genetic architecture of lint percentage but also facilitated designing high-yielding cotton through MAS breeding.

## 2. Results and Discussion

### 2.1. Large Variation in Lint Percentage in G. hirsutum Races

We evaluated the lint percentage of 189 accessions (188 accessions of *G. hirsutum* races and one cultivar (TM-1)) across five environments during 2014–2015 and 2015–2016. The phenotype of lint percentage showed continuous and extensive variations among the accessions under every individual environment (Table 1). The values ranged from 8.29 to 54.77%, with a mean value of 27.87% across the five environments. Moreover, the coefficients of variation (CV) exhibited relatively large values among the different environments, which ranged from 19.34 to 23.39%. However, the broad-sense heritability (*H*^2^) of the lint percentage was 77.20%. The correlation analysis of the lint percentage across the five environments ranged from 0.79 to 0.92, which uncovered significant and highly positive correlations among different environments (Table S1). The results suggested that lint percentage may have been controlled by multiple genes.

Several previous publications reported the evaluation of LP using the cultivated *G. hirsutum*. Song et al. [5], Su et al. [4], Sun et al. [8], Huang et al. [17], and Xing et al. [32] measured LP values of 276, 290, 719, 503, and 196 upland cotton accessions, respectively. The LP values ranged from 10.49 to 49.62%, from 28.49 to 47.01%, from 23.68 to 56.92%, from 27.07 to 47.38%, and from 22.1 to 49.9%, with the mean values of 37.60%, 40.89%, 39.85%, 38.29%, and 37.54%, respectively. The CV of LP were 9.60%, 7.08%, 7.62%, 7.93%, and 8.47%, respectively. In comparison with these studies, the LP value of *G. hirsutum* races is much lower than that of cultivated *G. hirsutum*, whereas the CV value of *G. hirsutum* races was higher, which implied that the LP of the cultivars was greatly improved but the genetic diversity rapidly reduced during the years of repeated selection in cotton yield breeding.

### 2.2. Genome-Wide Association Study for Lint Percentage

**Figure 1:**
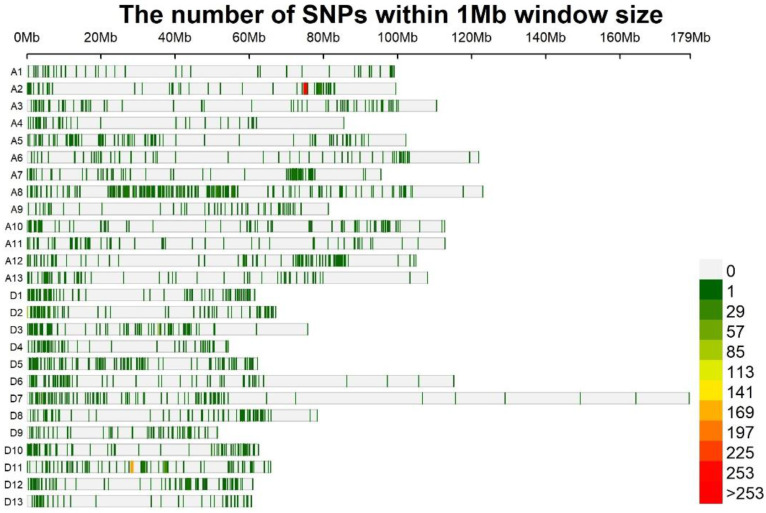
The distribution of markers related to lint percentage in *G. hirsutum*.

We carried out Genome-wide association study (GWAS) to discover loci underlying lint percentage across five environments using both the GAPIT 3.6.0 and Tassel 5 software. SNPs with −log_10_*p* values greater than 4.71 (−log_10_(1/51,268)) [18] were considered as significantly associated with lint percentage. Based on FaST-LMM, FarmCPU, BLINK, MLMM, and MLM of GAPIT 3.6.0 software and MLM of Tassel 5 software, we identified a total of 274 significantly associated SNPs randomly distributed on 24 chromosomes (Table S2) (these 274 SNPs were also identified significantly associated with lint percentage by GLM and super models of GAPIT 3.6.0 software, and GLM model of Tassel 5 software). Similarly, we summarized QTL-linkage and QTL-associated with lint percentage based on 54 previous reports and found the QTL were distributed unevenly across all the 26 chromosomes (Table S3; Figure 1), which meant that lint percentage was controlled by multiple genes.

Among the 274 SNPs, 45 were simultaneously identified at least in two environments or/and at least by two models (Table 2). These SNPs were randomly distributed on 18 chromosomes: A3–A5, A7, A8, A11–A13, D2–D5, and D8–D13. Specifically, four SNPs on chromosome A3 (TM6245, TM6246, TM6247, and TM6248) were located close together, and they were distributed in a 20 Kb region; ten SNPs on chromosome A11 were distributed at a distance of 1.86 Mb, of which nine SNPs (TM39097, TM39105, TM39111, TM39118, TM39119, TM39120, TM39121, TM39122, and TM39136) positioned in a 23 Kb region; two SNPs (TM55817 and TM55819) on chromosome D4; and two SNPs (TM57478 and TM57486) on chromosome D5 were distributed in 8.4 Kb and 248 Kb regions, respectively.

Of the 274 SNPs, 151 and 123 were positioned in the A and D sub-genomes, respectively; of the 45 SNPs simultaneously identified at least in two environments or/and at least by two models, 25 and 20 were distributed in the A and D sub-genomes, respectively. The results showed that the number of associated markers in the A sub-genome was not significantly different from that in the D sub-genome. Consistent with our opinion, in the previous reports, one GWAS identified eleven and eight associated SNPs in the A and D sub-genome [16], based on the genome resequencing of 318 accessions of core *G. hirsutum* germplasm; one GWAS detected eleven and ten lint-percentage-associated SNPs in the A and D sub-genomes, based on a CottonSNP63K array and a worldwide population consisting of 503 *G. hirsutum* accessions [17]; another GWAS identified nine and seven SNPs associated with lint percentage in the A and D sub-genomes, using SSR markers and 241 Upland cotton collections [33]. In addition, we surveyed 54 studies about linkage and association mapping on lint percentage (Table S3; Figure 1) and found that lint percentage QTL were distributed evenly between the A and D sub-genomes (1392 and 1285). Niu et al. [34] summarized QTL for lint percentage and found that the A sub-genome contained similar unique, tightly linked, and major QTL to that of the D sub-genome. Similarly, Said et al. [35] also reported that yield-related QTL were almost evenly distributed between the A and D sub-genomes [35]. The results suggested that the A and D sub-genomes contributed equally for lint percentage.

We also found that 220 (80.29%) and 54 (19.71%) SNPs were distributed as intergenic and intragenic; of the 45 SNPs detected at least in two environments or/and at least by two models, 36 (80%) and 9 (20%) were located as intergenic and intragenic (Table S4). The results suggested that most of the significant markers were positioned as intergenic. To confirm this, we analyzed the physical positions of the markers associated with lint percentage and the nearest makers of the QTL related, based on previous reports on QTL linkage and QTL associated with lint percentage, and found that most (1811, 87.57%) of the associated loci (totally 2068 SNPs investigated) were located as intergenic based on the genome sequence of the TM-1 (NAU v1.1) [36] (Table S3). The above results supported that most of the associated loci were distributed on non-protein-coding regions of the genome [37], which suggested that many loci implicated by GWAS might work through altering the genetic regulation of one or more target genes by regulating changes in target gene expression. Fortunately, transcriptome sequencing of the natural population could provide both the expression profile of each gene and the genetic variations in the gene coding regions.

### 2.3. Validation of the Stability of 45 SNPs Associated with Lint Percentage

**Figure 2:**
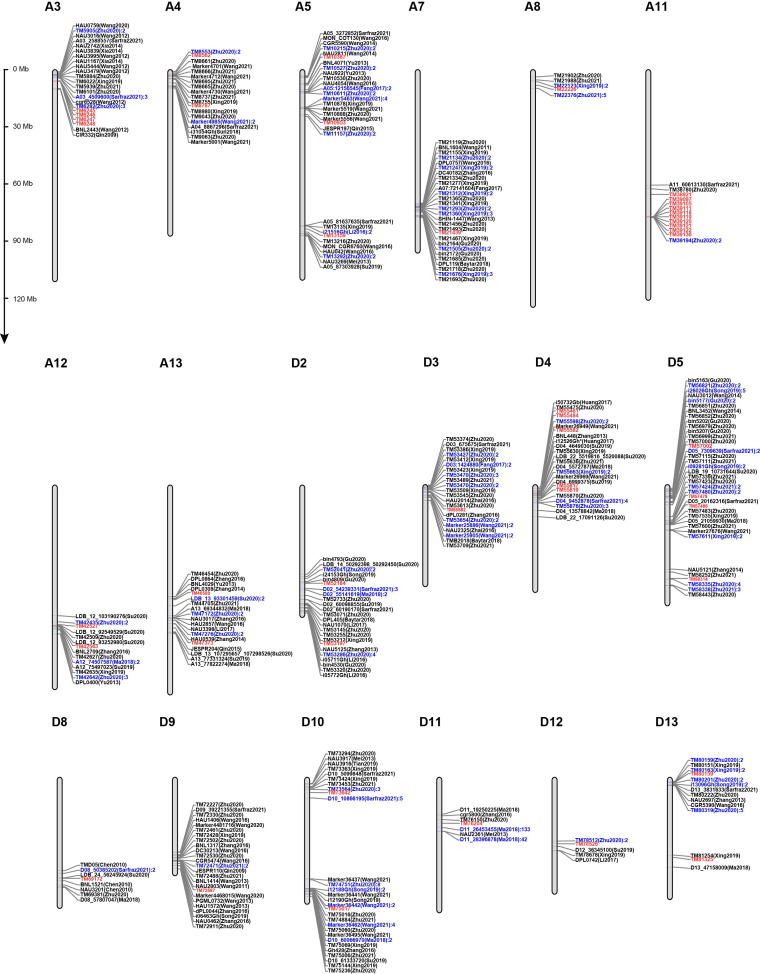
Comparison of the location of markers identified in previous and present studies. The markers marked red meant the markers associated with lint percentage identified in the present study; the markers marked blue meant there were at least two markers between the two markers marked up and down.

To validate the stability of these 45 significantly associated SNPs detected at least in two environments or by two models, we investigated 2677 markers/QTL for lint percentage mapped previously from 54 publications in upland cotton (Table S3). Then, the physical locations of 2831 markers were obtained through BLASTN [38], and 584 makers were shown being located within 5 Mb up- and downstream of the 45 SNPs (Figure 2). In addition, fifteen SNPs identified in this study (TM6245, TM6246, TM6247, TM6248, TM21439, TM42563, TM53197, TM53588, TM55461, TM555582, TM57478, TM57486, TM73642, TM75017, and TM80159), were localized within or near previously reported markers/QTLs. Specifically, four SNPs (TM6245, TM6246, TM6247, and TM6248) on A3 were located on the overlap of *qLP-C-3* (CGR6528–BNL2443) detected by Wang et al. [39] and were 100 Kb away from TM6282 detected by Zhu et al. [13]. Eight SNPs (TM21439 on A7, TM42563 on A12, TM53197 on D2, TM53588 on D3, TM55461 on D4, TM55582 on D4, TM57478 and TM57486 on D5) were within 200 Kb away from TM21493 detected by Zhu et al. [13], LDB_12_93252980 detected by Su et al. [4], TM53255 detected by Zhu et al. [13], TM53212 detected by Xing et al. [32], DPL0281 detected by Zhang et al. [26], SWU20808 detected by Yang et al. [40], TM55475 detected by Zhu et al. [13], Marker26949 detected by Wang et al. [41], TM57480 and TM57483 detected by Zhu et al. [13], or D05_20162316 detected by Sarfraz et al. [20]. TM73642 on D10 was 70 Kb away from TM73693 detected by Zhu et al. [13]. TM75017 on D10 also was approximately 70 Kb away from i12188Gh, i12189Gh, and i12190Gh detected by Song et al. [5] and was 70 Kb and 150 Kb away from *qLP-Dt10-3* (Marker36441—Marker36437) and *qLP-Dt10-2* (Marker36463—Marker36442) detected by Wang et al. [41], respectively. TM80159 on D13 was 34–170 Kb away from three SNPs (TM80151, TM80163, and TM80171) detected by Xing et al. [32]. The results above suggested that the 45 SNPs corresponded to markers/QTL reported previously. These markers were repeatedly detected in different populations with different genetic backgrounds and could potentially be considered in the MAS of target traits.

### 2.4. Discovery of Candidate Genes for Lint Percentage

**Figure 3:**
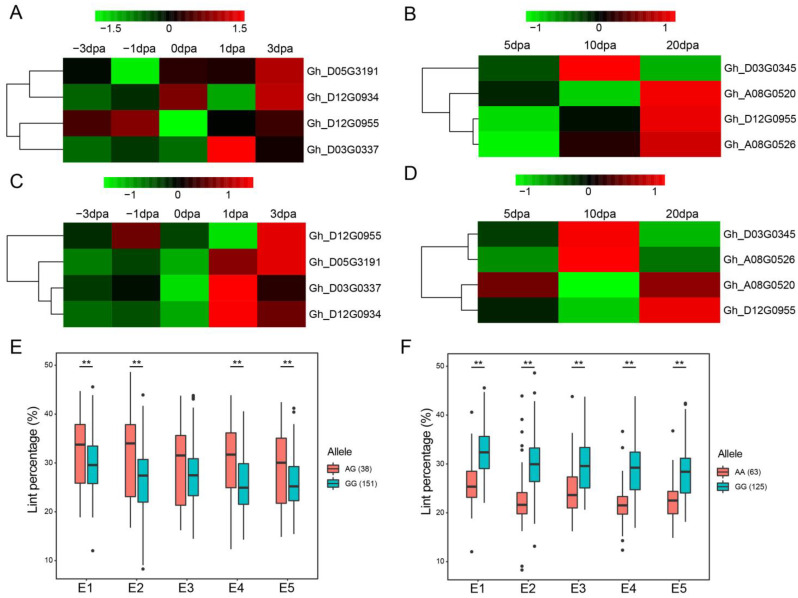
Gene expression pattern of the candidate genes of lint percentage. (**A**) The expression pattern of candidate genes detected by RNA-seq in the ovule tissue. (**B**) The expression pattern of candidate genes detected by RNA-seq in the fiber tissue. (**C**) The expression pattern of candidate genes detected by qRT-PCR in the ovule tissue. (**D**) The expression pattern of candidate genes detected by qRT-PCR in the fiber tissue. (**E**) Box plots for lint percentage of TM78526. (**F**) Box plots for lint percentage of TM22226. “**”, significant at a = 0.01 level. “.”, the outliers.

In total, 11 of the 45 SNPs were identified at least in two environments. We compared the lint percentage between alleles of the eleven associated SNPs in each environment and found that significant differences existed between the superior and inferior alleles (Table S5; Figure 3). The superior alleles might be integrated properly by marker-assisted selection in order to improve lint percentage. We then discovered the candidate genes near these eleven associated SNPs, based on available genes’ annotation information of the *G. hirsutum* TM-1 genome [36]. A total of 335 candidate genes was obtained from the 550 Kb up- and downstream regions of 11 SNPs. Based on RNA-seq data of the ovule (including −3, −1, 0, 1, and 3 dpa) and fiber (including 5, 10, and 20 dpa) tissues in TM-1 [36], we predicted the candidate genes involved in fiber initiation and fiber elongation, respectively. After removing the genes with FPKM < 2, the gene expression changed within twofold, and the homolog without annotation, 125 genes (including 80 for the ovule and 86 for the fiber, 41 genes were overlapped in both ovule and fiber) was retained for further analysis (Figure S1; Tables S6 and S7). Based on the gene annotation information of these 125 genes in Arabidopsis, eleven genes were found to be related to root development; eight genes were related to plant-type cell wall biogenesis; six genes were related to auxin polar transport or its signaling pathway; five genes were related to flower development and response to abscisic acid, respectively; four genes were related to cell differentiation and cell division, respectively; three genes were related to plant epidermis development, pollen maturation, anther dehiscence, and cell tip growth, respectively; and two genes were related to anther development, pollen development, pollen tube, and unidimensional cell growth, respectively (Tables S6 and S7).

The gene expression patterns of these 125 genes were reflected (Figure S1) using RNA-seq data of TM-1 [36]. For 80 genes related to the ovule tissues, twenty-one and eight genes were expressed highly at −3 dpa and −2 dpa, which may have played roles in the differentiation of fiber cells; nine genes had high gene expressions at 0 dpa, which may been involved in producing fiber cell initials; 13 genes were expressed highly at 1 dpa, which may participated in the transformation from non-polar expansion to polar elongation; 14 genes expressed strongly at 3 dpa, which may participated in cotton fiber elongation. For 86 genes related to the fiber tissues, fourteen, eight, and ten genes, were expressed highly at 5 dpa, 10 dpa, and both stages, respectively, which may been involved in cell elongation; 41 genes were expressed highly at 20 dpa, suggesting that these genes may have functioned in cell elongation and/or primary wall biosynthesis.

Referring to gene annotation and the RNA-seq analysis, we selected several genes to perform qRT-PCR using ovule (−2, −1, 0, 1, and 3 dpa) and fiber (5, 10, and 20 dpa) tissues. The results showed that the expression patterns of most genes determined by RNA-seq and qRT-PCR were similar (Figure 3). In the ovule tissues, *Gh_D05G3191* and *Gh_D12G0955* were highly expressed at 3 dpa; *Gh_D03G0337* and *Gh_D12G0934* were highly expressed at 1 dpa. Of these four genes mentioned above, *Gh_D12G0934* was found to be involved in the progress of cell differentiation, cell division, circadian rhythm, regulation of transcription, DNA-templating, and root hair cell tip growth. The occurrence of root hair was similar to fiber initiation, suggesting that *Gh_D12G0934* might have been involved in fiber initiation. In the fiber tissues, *Gh_D03G0345* was strongly expressed at 10 dpa, *Gh_A08G0526* was highly expressed from 10 dpa to 20 dpa, whereas *Gh_A08G0520* and *Gh_D12G0955* were preferentially expressed at 20 dpa. Among these four genes, *Gh_A08G0526* was related to the progress of plant-type cell wall biogenesis, regulation of transcription, DNA-templating, and shoot system development. We predicted that *Gh_A08G0526* might function in secondary wall deposition of cotton fiber.

Genes control phenotypes, and the identifications and isolations of the genes regulating LP are essential for MAS and gene engineering breeding. Through GWAS for lint percentage, fruitful key candidate genes have been yielded directly based on linkage disequilibrium (Table S8), such as *Gh_A02G1268* (*MIPS*, a member of the myo-inositol-1-phosphate synthase gene family) [42]; *WD40* [15]; *Gh_D08G2376* (a homolog of *AT3G07020* and *GhSGT1*) [17]; *Gh_A02G1392* (*AIL6*, a homolog of the AP2/ETHYLENE RESPONSE FACTOR (ERF)-type transcription-factor-encoding gene AINTEGUMENTA-like 6 in Arabidopsis) and *Gh_D08G0312* (*EIL*, encoding ethylene insensitive 3-like family protein) [16]; *Gh_D03G1064* (*FRI*, encoding a FRIGIDA-like protein) and *Gh_D12G2354* (encoding a GPR107-like protein) [8]; *Gh_D02G0025* (encoding tetratricopeptide repeat (TPR)-like superfamily protein) [19]; *Gh_D05G1124* (encoding Protein phosphatase 2C family protein) and *Gh_D05G0313* (the ortholog of *AtLUT2*) [5]; *Gh_A02G1269* (encoding chaperone protein dnaJ 20), *Gh_A02G1278* (encoding E3 ubiquitin-protein ligase RHA2A), *Gh_A02G1280*, and *Gh_A02G1295* (*AtAMP1*, ALTERED MERISTEM PROGRAM1 in Arabidopsis) [43]; *Gh_A05G2488* (a member of auxin transport facilitator family named PIN-FORMED LIKES proteins (PILS)), *Gh_D13G0342* (RAB GTPase homolog G3F (RABG3F)), and *Gh_A10G2138* (*PRA1*, encoding prenylated RAB acceptor protein 1) [13]; and *GH_A07G1389* (encoding a tetratricopeptide repeat (TPR)-like superfamily protein) [44]. According to the prediction of gene function, transcription; signaling pathways (such as hormone, calcium (Ca_2_^+^), and MAPK); energy metabolism (such as starch and sucrose metabolism); substance transport; cell division; and cell wall development are important biological processes for LP formation.

### 2.5. Gh_A08G0526 and Gh_D12G0934 Were Candidate Genes for Lint Percentage

**Figure 4:**
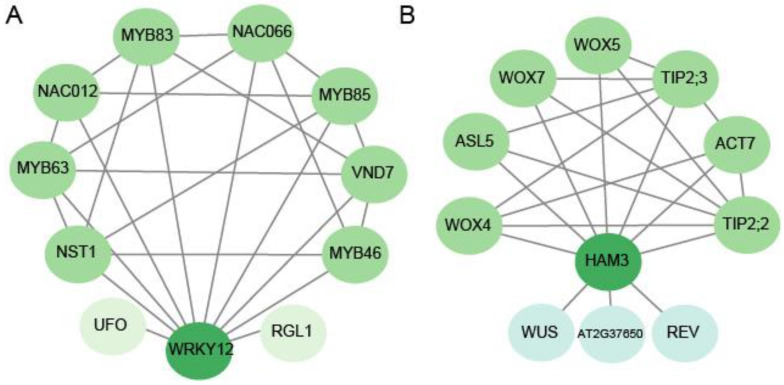
Interaction network of these two key candidate genes in Arabidopsis. (**A**) Interaction network of *Gh_A08G0526*. (**B**) Interaction network of *Gh_D12G0934*.

We separately investigated the protein interaction of *Gh_A08G0526* and *Gh_D12G0934*. Until now, there has been no database available to predict protein interactions of genes in cotton directly. Here, we predicted the protein interactions of the homologous genes in Arabidopsis (Figure 4). For *Gh_A08G0526*, its homolog in Arabidopsis is *AT2G44745.1* (*WRKY12*), which is involved in plant-type cell wall biogenesis, regulation of transcription, DNA-templateing, and/or shoot system development. Ten interactions protein of *WRKY12* (*AT2G44745.1*) were identified, and a total of twenty-five protein interactive relationships was identified between these eleven genes (Table S9), of which four (*AT1G79180.1*, *AT5G12870.1*, *AT4G22680.1*, and *AT3G08500.1*, homologous with *Gh_D08G2456*, *Gh_D09G1082*, *Gh_D09G1690*, and *Gh_A08G1308*, respectively) and two (*AT3G61910.1* and *AT1G32770.1* homologous with *Gh_D11G1062*, *Gh_A11G0915*, and *Gh_D12G2359*, respectively) genes were members of the *MYB* and *NAC* family, respectively. The interacting genes included *MYB63*, *MYB83*, *MYB85*, *MYB46*, *NAC012*, *NAC066*, *NST1*, and *VND7*. The related genes in cotton were reported mainly to be involved in fiber initiation, fiber elongation, and/or secondary wall deposition, then contributing to the formation of lint. For instance, STV106, an NAC-domain-containing protein, could regulate secondary wall biogenesis [45]. *GhMYB212* regulated the gene expression pattern of *GhSWEET12*, and further played an important role during the fiber elongation; plants with silenced *GhMYB212* could accumulate less sucrose and glucose during the development of fiber and produce shorter fibers and lower lint yields [46]. *R2R3 MYB* (*GhMYB25-like*) RNAi led to fiberless seeds, whereas trichomes elsewhere were normal [47]; *GhMYB25*-silenced cotton altered the timing of fiber elongating rapidly, resulting in the short fibers and trichomes of other tissues reducing dramatically and, finally, reducing the seed production [48]. *GhMYB7*-overexpressed cotton plants accelerated the cellulose biosynthesis of the secondary cell walls and resulted in shorter fibers with thicker walls, but *GhMYB7* RNAi plants delayed cellulose synthesis and produced longer fibers with thinner walls [49]. In Arabidopsis, XND1 could regulate the activity of NST1 during the formation of the secondary cell walls of fiber cells [50]. *GhFSN1*, as a positive regulator, activated its downstream genes involved in the secondary cell wall and further controlled the secondary cell wall formation of cotton fibers [51]. The critical genes related to the formation of the secondary cell wall, such as the transcription factor gene *MYB46* and its homolog *MYB83*, could directly be regulated by *VND7* [52,53,54]. The overexpression of *MYB46* or *MYB83* led to the secondary cell walls thickening, whereas the *myb46* and *myb83* loss-of-function double mutant did not yield any observable secondary cell walls [55]. *GhWRKY16* functioned as a positive regulator during fiber initiation and elongation, silencing *GhWRKY16* in cotton, produced shorter fibers and reducing the number of fiber protrusions on the ovule [56]. Taken together, NAC domain transcription factors function as master switches during the thickening of the secondary cell wall; MYB transcription factors, downstream from the *NST* genes, are also important in secondary wall biosynthesis [26,45]. The above results suggested that *Gh_A08G0526* might be involved in fiber development through regulating MYB and NAC.

For *Gh_D12G0934*, its homolog in Arabidopsis *AT4G00150.1* (*HAM3*), is involved in cell differentiation, cell division, circadian rhythm, regulation of transcription, DNA-templating, and/or root hair cell tip growth. Ten pairs of HAM3 (AT4G00150.1) protein interactions were identified, and a total of twenty-one protein interactive relationships was identified between these eleven genes (Table S10), of which three (*AT3G11260.1*, *AT1G46480.1*, and *AT5G05770.1*, homologous with *Gh_A10G2087*, *Gh_D05G1962*, and *Gh_A10G2087*, respectively) and two genes (*AT4G17340.1* and *AT5G47450.1*, homologous with *Gh_Sca059366G01* and *Gh_D03G1568*, respectively) were also family members of the *WOX* and *TIP*. These related genes functioned as being related to root development. For instance, ACT7 participated in root growth, epidermal cell specification, cell division, and root architecture [57]; the *act7-4* mutant reduced the root growth, twisted root apical cells, and obliqued junctions between cells dramatically [58]. *WOX4* functioned to facilitate the differentiation and/or maintenance of the initial cells of the developing vasculature; silencing *WOX4* in Arabidopsis-generated small plants, with differentiated xylem and phloem reducing severely [59]. The expressions of *WOX11/12* and *WOX5/7* were vital for the initiation of the root primordium during the formation of root tissue in Arabidopsis [60]. The lateral roots of the *35S: AtASL5* transgenic cockscombs had a central–peripheral defect [61]. *Atham1,2,3* mutant plants generated significantly smaller root meristems in both the longitudinal and radial axes than those of a wild type by reducing the division rates of root meristem cells [62].

Furthermore, we analyzed the *cis*-elements of the promoter region (2000 bp sequence upstream of the coding sequence) for the two candidate genes (*Gh_A08G0526* and *Gh_D12G0934*) and found that seven elements were related to light in the promoter regions of *Gh_A08G0526* and *Gh_D12G0934*, respectively (Table S11). An auxin-responsive element (TGA-element) was also identified in the promoter region of *Gh_D12G0934*. Moreover, we identified miRNAs targeting *Gh_D12G0934* in *G. hirsutum* and found that ghr-miR171 targeted *Gh_D12G0934*. In cotton, *miR171* showed significantly higher expression in the fuzzless–lintless mutant Xu-142-fl than in the wildtype [63]. Meanwhile, *miR171* was expressed much more abundantly in the ovule during the initiation than in fiber during elongation and secondary wall thickening in 3–79 [64]. Generally speaking, the root morphogenesis had a similar mechanism to the cotton fiber initiation; therefore, *Gh_D12G0934* might have affected lint percentage by controlling fiber initiation. However, its function needs to be verified by transgenic technologies, such as gene overexpression and RNA silencing. The above results suggested that *Gh_A08G0526* and *Gh_D12G0934* might be crucial candidate genes involved in fiber elongation or initiation.

## 3. Materials and Methods

### 3.1. Natural Population Germplasm and Lint Percentage Evaluation

A natural population, including 189 accessions of *G. hirsutum*, was used for association mapping in the present study. One-hundred-and eighty-eight accessions consisted of all the seven *G. hirsutum* races, and the other was a representative of the cultivars, the upland cotton genetic standard *G. hirsutum* L. acc. Texas Marker-1 (TM-1) [30]. All the *G. hirsutum* races were originally introduced from the USDA-ARS Southern Agricultural Research Center in College Station, Texas, USA, were perennially purified and preserved in the National Wild Cotton Nursery, Sanya, Hainan, China, and were supervised by the Institute of Cotton Research of Chinese Academy of Agricultural Sciences (ICR-CAAS), Anyang, Henan, China. All the accessions were legally planted in five environments (E_1_: 2014–2015 Yacheng; E_2_: 2014–2015 Damao; E_3_: 2015–2016 Yacheng; E_4_: 2015–2016 Damao in the greenhouse; E_5_: 2015–2016 Damao). In each experimental environment, 20–25 plants were planted in a single row plot (the row length and the row interval were 5.0 m and 1.0 m, respectively). Standard local field management practices were performed throughout the whole planting season.

At the open-boll bloom stage, we randomly collected 30 naturally full open bolls from the middle of each row and weighted the seed–cotton yield of each sample. After ginning, the fiber yield was weighted, and the lint percentage was calculated based on the ratio of the lint-to-seed–cotton weight. Then, we used R 4.1.2 software to carry out the descriptive statistical analysis, the correlation analysis, the best linear unbiased prediction (BLUP) of each accession, and *H*^2^ calculation of lint percentage [65,66].

### 3.2. SNP Genotyping and Population Structure Assessment

Detailed descriptions of the SNP genotyping processes were published previously [30]. In brief, high-quality genomic DNA of each accession was extracted from the young leaf tissue and was diluted to 50 ng/μL. All the 189 accessions were genotyped by the CottonSNP80K chip. The SNP data were analyzed according to GenomeStudio Genotyping software (v1.9.4, Illumina, San Diego, CA, USA) and then filtered with a minor allele frequency (MAF) < 0.05, integrity < 50%, or call rate < 90%. Finally, a total of 51,268 high-quality SNPs were obtained and were used for further analysis, including population structure, the kinship coefficient of every pair of accessions, linkage disequilibrium (LD) analysis, and genome-wide association mapping.

### 3.3. Genome-Wide Association Study of Lint Percentage

Genome-wide association study for lint percentage was conducted using GAPIT 3.6.0 [67] and Tassel 5.0 [68] software. For GAPIT 3.6.0 software, we selected seven models, including GLM, SUPER, MLM, Fast-LMM, FarmCPU, BLINK, and MLMM; the PCA.total was set as two. For Tassel 5.0 software, we used GLM and MLM models. The significant threshold for the marker–trait associations was set as *p* = 1/*n* (where *n* = 51,268, the number of SNPs totally used for GWAS, −log_10_ *p* = 4.71) [18]. To validate the stability of the associated SNPs, we investigated markers/QTL for lint percentage from previously publications in upland cotton. Based on the TM-1 reference genome [36], the physical locations of markers were obtained by BLASTN [38] and then were compared with the physical location of the SNPs detected in this study. For the SNPs detected in two or more environments, the phenotypic values of every haplotype/allele were analyzed by the two-tailed *t*-tests using SAS 9.4 software (SAS Institute Inc., Cary, NC, USA).

### 3.4. Prediction and Identification of Related Candidate Gene

Based on gene annotation and gene expression pattern of the genes in the *G. hirsutum* acc. TM-1 genome [36], we predicted the candidate genes within an LD (550 Kb) up- and downstream of significantly associated SNPs detected in more than one environment. Firstly, we obtained the raw RNA-seq data of TM-1 tissues (including ovule of −3, −1, 0, 1, and 3 days post-anthesis (dpa); and fiber of 5, 10, and 20 dpa) from NCBI Sequence Read Archive (accession no. PRJNA248163). Then, we carried out the gene expression through TopHat 2.1.1 and Cufflinks 2.2.1 software [69] and extracted the normalized FPKM values as the gene expression levels. Secondly, we filtered the genes with FPKM < 2 in ovule or in fiber, and deleted the genes with gene expression changed within twofold. Thirdly, we picked up the homologous genes in Arabidopsis, searched their annotation from the database of the Arabidopsis information resource, and removed the genes without annotation. According to the annotation, the candidate genes of lint percentage were explored.

The expression of the candidate genes was further analyzed by qRT-PCR. Using EASYspin plus plant RNA mini kit (RN38, Aidlab, Beijing, China),we extracted high-quality total RNA from TM-1 tissues, including ovules at −2, −1, 0, 1, and 3 dpa and fibers at 5, 10, and 20 dpa, and then reverse-transcribed into cDNA following the protocol guidelines of GoScript™ Reverse Transcription System (A5001, Promega, Madison, WI, USA) with a 20 μL RT reaction: 5 μL GoScript^TM^ 5* Reaction buffer, 5 μL 25 mM MgCl_2_, 2 μL PCR Nucleotide Mix, 1.0 μL GoScript^TM^ Reverse Transcriptase, 0.5 μL Recombinant RNasin Ribonuclease inhibitor, 0.5 μL 500 μg/mg Oligo(dT)_15_Primer, 0.5 μL Random Primers, and 1 μg total RNAs and nuclease-free water. After pre-denaturation at 70 °C for 10 min, RNAs turned to the ice, then the RT reaction was performed following the temperature program: 5 min at 25 °C, 60 min at 42 °C, 15 min at 70 °C, and was kept at −20 °C. qRT-PCR amplification for these candidate genes was implemented using Power SYBR Green PCR Master Mix (4309155, Applied Biosystems, Foster City, CA, USA) on a QuantStudioTM 6 Flex 384-well system (Applied Biosystems™, Foster City, CA, USA). Specifically, the qRT-PCR was performed in a 20 μL reaction, including 1 μL diluted cDNA, 10 μL SyberGreen PCR Master Mix, 2 μL primers, and nuclease-free water. The temperature program was 10 min at 95 °C, 45 cycles of 15 s at 95 °C, and 60 s at 60 °C. The *G. hirsutum actin* gene *Ghactin7* (*LOC107959437*) was the internal reference [70], its primer and the specific primers of target genes were listed in Table S12. The expression pattern of candidate genes were analyzed using the comparative 2^−ΔΔCt^ method [71] as follows: −ΔΔCt = [(C_T gene of interest_ − C_T internal control_) sample A − (C_T gene of interest_ − C_T internal control_) sample B]. For the ovule tissues, one biological replicate of ovules at −2 dpa was sample B; while, for the fiber tissues, one biological replicate of fibers at 5 dpa was sample B. Finally, the heatmaps of the candidate gene expression patterns for lint percentage were produced with Mev 4.9 software [72].

### 3.5. The Protein–Protein Interaction Analysis, the Cis-Elements, and the Predicted miRNA Targeting the Candidate Genes

We imported the gene IDs of homologous candidate genes in Arabidopsis to the STRING database [73] and obtained the interaction network information of the candidate genes in Arabidopsis. Then, the interaction network was visualized using Cytoscape 3.7.2 [74]. Finally, the Arabidopsis interaction protein IDs were changed to *G. hirsutum* IDs for the prediction of the interaction proteins in *G. hirsutum.*

To predict the possible cis-acting elements of the crucial candidate genes, the 2000 bp upstream region of the candidate genes, considered as the promoter region, was extracted and delivered to PlantCARE [75]. The statistics of predicted cis-acting elements were compiled by category.

Furthermore, the miRNA database of the *G. hirsutum* (v1.1) was downloaded from the plant microRNA database [76] and was delivered to the psRNATarget to investigate the potential miRNAs of the candidate genes [77].

## 4. Conclusions

A genome-wide association study was conducted, using 188 accessions of *G. hirsutum* races and TM-1. Forty-five SNPs were identified at least in two environments or/and at least with two models, which were located near 584 markers/QTL related to lint percentage identified in previous publications. Eleven SNPs were detected as significantly associated with lint percentage at least in two environments, and their 550 Kb (a linkage disequilibrium) up- and downstream region contained 335 genes. Based on RNA sequencing, qRT-PCR, gene annotations, protein–protein interaction networks, the *cis*-elements of the promotor region, and the related miRNA predictions, *Gh_D12G0934* and *Gh_A08G0526* were determined as crucial candidate genes for fiber initiation and elongation, respectively. These SNPs and candidate genes identified loci/genes for analyzing the genetic architectures of lint percentages in *G. hirsutum.*

## Figures and Tables

**Table 1:** Descriptive statistical analysis of lint percentage of *G. hirsutum* races across five environments.

Env.	Max	Min	Mean	Std.	CV	Skewness	Kurtosis
E_1_	47.55	12.03	30.31	5.86	19.34	0.14	−0.15
E_2_	54.77	8.29	28	6.55	23.39	0.43	0.85
E_3_	43.8	14.47	28.04	6.21	22.14	0.48	−0.24
E_4_	43.86	12.35	26.61	6.21	23.34	0.39	−0.39
E_5_	42.43	14.87	26.37	5.76	21.83	0.51	−0.03

E_1_, E_2_, E_3_, E_4_, and E_5_ indicate the five environments: 2014–2015 Yacheng, 2014–2015 Damao, 2015–2016 Yacheng, 2015–2016 Damao in the greenhouse, and 2015–2016 Damao, respectively; CV, coefficient of variation.

**Table 2:** SNPs detected significantly associated with lint percentage at least in two environments or at least by two models.

SNP	Chr	Pos. (bp)	GAPIT					Tassel
			FaST-LMM	FarmCPU	BLINK	MLMM	MLM	TMLM
TM6245	A3	6,000,457	BLUP (4.90)			BLUP (4.90)		
TM6246	A3	6,008,064	BLUP (5.10)		BLUP (6.99)	BLUP (5.10)		
TM6247	A3	6,015,363	BLUP (5.10)			BLUP (5.10)		
TM6248	A3	6,020,227	BLUP (4.90)			BLUP (4.90)		
TM8562	A4	1,704,160		E_5_ (6.37)	E_5_ (6.23)			
TM8787	A4	5,125,340		E_1_ (7.94)	E_1_ (10.43)			
TM10387	A5	7,465,603	E_5_ (4.71)			E_5_ (4.71)		
TM10953	A5	21,122,062		E_3_ (4.97)	E_3_ (9.45)			
TM13159	A5	83,646,730		E_3_ (6.75); E_4_ (6.30); E_5_ (6.66)	E_3_ (17.39); E_4_ (6.90); E_5_ (9.84)			
TM21439	A7	73,820,001	E_2_ (5.42)			E_2_ (5.42)		
TM22226	A8	8,180,911		E_4_ (15.51)	E_4_ (11.19); E_5_ (6.70)			
TM38921	A11	65,498,934		E_2_ (6.45)	E_2_ (7.92)			
TM39097	A11	77,128,215					E_2_ (5.41)	E_2_ (5.01)
TM39105	A11	77,162,385					E_2_ (4.94)	E_2_ (5.23)
TM39111	A11	77,201,721					E_2_ (4.85)	E_2_ (5.06)
TM39118	A11	77,245,152					E_2_ (5.41)	E_2_ (4.73)
TM39119	A11	77,250,629	E_2_ (6.57)	E_2_ (7.05)	E_2_ (4.99)	E_2_ (6.57)	E_2_ (5.48)	E_2_ (4.80)
TM39120	A11	77,254,990					E_2_ (5.35)	E_2_ (5.42)
TM39121	A11	77,259,287					E_2_ (5.39)	E_2_ (4.72)
TM39122	A11	77,267,198					E_2_ (5.33)	E_2_ (5.42)
TM39136	A11	77,356,582		E_3_ (9.26); E_5_ (12.70)	E_1_ (5.59); E_2_ (4.84); E_3_ (14.49); E_4_ (5.33); E_5_ (13.96)			
TM42527	A12	73,222,779		E_1_ (5.91)	E_1_ (13.91)			
TM42563	A12	73,856,986		E_2_ (5.34)	E_2_ (7.14)			
TM46588	A13	60,134,206		E_1_ (6.86)	E_1_ (14.22)			
TM47373	A13	72,835,782		E_3_ (8.73)	E_3_ (7.06)			
TM52184	D2	51,543,776		E_1_ (4.89); E_4_ (10.43)	E_4_ (6.60)			
TM53197	D2	64,206,361		E_1_ (4.77)	E_3_ (6.30)			
TM53588	D3	4,016,585		E_4_ (15.33)	E_1_ (13.39); E_4_ (14.39); E_5_ (10.63)			
TM55461	D4	2,087,134	E_2_ (6.19)		E_2_ (9.29)	E_2_ (6.19)	E_2_ (5.23)	
TM55484	D4	2,902,882		E_5_ (9.59)	E_5_ (13.00)			
TM55582	D4	3,824,554		E_2_ (6.80)				E_2_ (6.86)
TM55817	D4	7,622,861	E_2_ (5.35)			E_2_ (5.35)		
TM55819	D4	7,631,258	E_2_ (5.35)			E_2_ (5.35)		
TM57002	D5	6,810,289						E_1_ (4.85); E_3_ (5.82)
TM57478	D5	19,820,229	E_4_ (4.95)	E_4_ (22.33); E_5_ (4.79)	E_4_ (12.47)	E_4_ (4.95)	E_4_ (4.76)	
TM57486	D5	20,068,395		E_2_ (5.45)				E_3_ (4.87)
TM58314	D5	49,232,321						E_1_ (4.90); E_3_ (4.77)
TM69172	D8	53,049,470		E_3_ (13.74)	E_3_ (8.17)			
TM72587	D9	43,752,600		E_3_ (7.45)	E_3_ (7.83)			
TM73642	D10	8,030,303		E_3_ (5.42)	E_3_ (6.93)			
TM75017	D10	58,300,092		E_4_ (4.72)	E_4_ (13.12)			
TM76204	D11	24,111,532		E_3_ (8.00)	E_3_ (14.52)			
TM78526	D12	34,893,826	E_2_ (4.84)	E_3_ (5.16)		E_2_ (4.84)		
TM80159	D13	2,590,468		E_5_ (5.39)	E_5_ (9.39)			
TM81325	D13	42,144,468		E_1_ (7.99); E_2_ (9.96)	E_1_ (6.54)			

E_1_, E_2_, E_3_, E_4_, and E_5_ indicate the five environments 2014–2015 Yacheng, 2014–2015 Damao, 2015–2016 Yacheng, 2015–2016 Damao in the greenhouse, and 2015–2016 Damao, respectively; Fast-LMM, FarmCPU, BLINK, MLMM, and MLM were models used in GAPIT 3.6.0 software; TMLM 5 was the model used in Tassel 5 software. All the SNPs were also detected by GLM and SUPER in GAPIT 3.6.0 software and were also identified by GLM of Tassel 5 software.

## Data Availability

Not applicable.

## Supplementary Materials

The supporting information can be downloaded at: https://www.mdpi.com/article/10.3390/ijms241210404/s1.
