# Supplementary material for: Genome-Wide Association Study of Lint Percentage in Gossypium hirsutum L. Races
Source: Int J Mol Sci. 2023 Jun 20;24(12):10404. doi: 10.3390/ijms241210404 (PMC10299271; doi:10.3390/ijms241210404)
Supplement: Supplementary file 1 [file ijms-24-10404-s001.zip › Supplementary Figure S1.pdf]

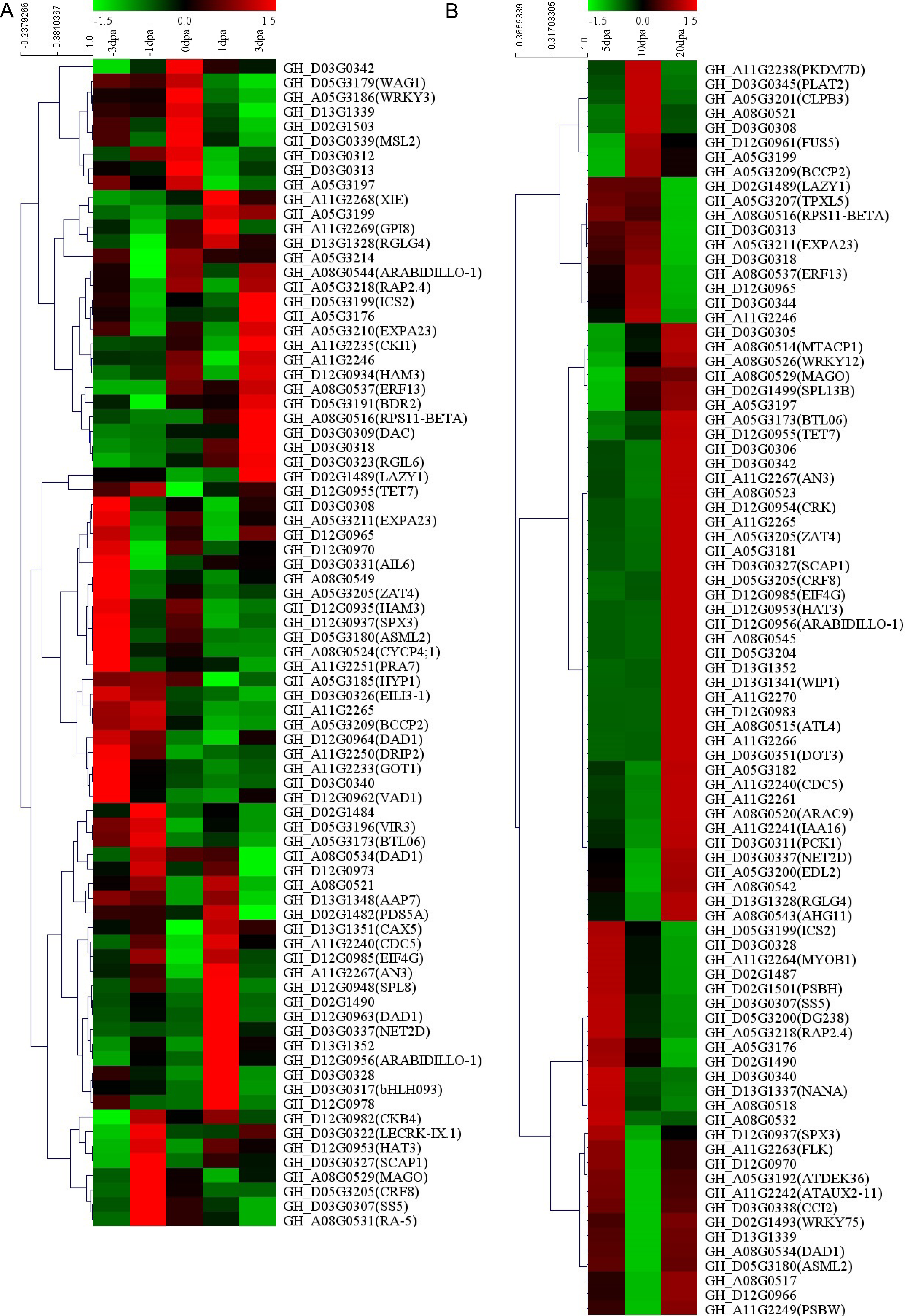
A
-0.2379266
0.3810367
1.0
-1.5
0.0
1.5
-3dpa
-1dpa
0dpa
1dpa
3dpa
GH_D03G0342
GH_D05G3179(WAG1)
GH_A05G3186(WRKY3)
GH_D13G1339
GH_D02G1503
GH_D03G0339(MSL2)
GH_D03G0312
GH_D03G0313
GH_A05G3197
GH_A11G2268(XIE)
GH_A05G3199
GH_A11G2269(GPI8)
GH_D13G1328(RGLG4)
GH_A05G3214
GH_A08G0544(ARABIDILLO-1)
GH_A05G3218(RAP2.4)
GH_D05G3199(ICS2)
GH_A05G3176
GH_A05G3210(EXPA23)
GH_A11G2235(CKI1)
GH_A11G2246
GH_D12G0934(HAM3)
GH_A08G0537(ERF13)
GH_D05G3191(BDR2)
GH_A08G0516(RPS11-BETA)
GH_D03G0309(DAC)
GH_D03G0318
GH_D03G0323(RGIL6)
GH_D02G1489(LAZY1)
GH_D12G0955(TET7)
GH_D03G0308
GH_A05G3211(EXPA23)
GH_D12G0965
GH_D12G0970
GH_D03G0331(AIL6)
GH_A08G0549
GH_A05G3205(ZAT4)
GH_D12G0935(HAM3)
GH_D12G0937(SPX3)
GH_D05G3180(ASML2)
GH_A08G0524(CYCP4;1)
GH_A11G2251(PRA7)
GH_A05G3185(HYP1)
GH_D03G0326(EILI3-1)
GH_A11G2265
GH_A05G3209(BCCP2)
GH_D12G0964(DAD1)
GH_A11G2250(DRIP2)
GH_A11G2233(GOT1)
GH_D03G0340
GH_D12G0962(VAD1)
GH_D02G1484
GH_D05G3196(VIR3)
GH_A05G3173(BTL06)
GH_A08G0534(DAD1)
GH_D12G0973
GH_A08G0521
GH_D13G1348(AAP7)
GH_D02G1482(PDS5A)
GH_D13G1351(CAX5)
GH_A11G2240(CDC5)
GH_D12G0985(EIF4G)
GH_A11G2267(AN3)
GH_D12G0948(SPL8)
GH_D02G1490
GH_D12G0963(DAD1)
GH_D03G0337(NET2D)
GH_D13G1352
GH_D12G0956(ARABIDILLO-1)
GH_D03G0328
GH_D03G0317(bHLH093)
GH_D12G0978
GH_D12G0982(CKB4)
GH_D03G0322(LECRK-IX.1)
GH_D12G0953(HAT3)
GH_D03G0327(SCAP1)
GH_A08G0529(MAGO)
GH_D05G3205(CRF8)
GH_D03G0307(SS5)
GH_A08G0531(RA-5)
B
-0.3659339
0.31703305
1.0
-1.5
0.0
1.5
5dpa
10dpa
20dpa
GH_A11G2238(PKDM7D)
GH_D03G0345(PLAT2)
GH_A05G3201(CLPB3)
GH_A08G0521
GH_D03G0308
GH_D12G0961(FUS5)
GH_A05G3199
GH_A05G3209(BCCP2)
GH_D02G1489(LAZY1)
GH_A05G3207(TPXL5)
GH_A08G0516(RPS11-BETA)
GH_D03G0313
GH_A05G3211(EXPA23)
GH_D03G0318
GH_A08G0537(ERF13)
GH_D12G0965
GH_D03G0344
GH_A11G2246
GH_D03G0305
GH_A08G0514(MTACP1)
GH_A08G0526(WRKY12)
GH_A08G0529(MAGO)
GH_D02G1499(SPL13B)
GH_A05G3197
GH_A05G3173(BTL06)
GH_D12G0955(TET7)
GH_D03G0306
GH_D03G0342
GH_A11G2267(AN3)
GH_A08G0523
GH_D12G0954(CRK)
GH_A11G2265
GH_A05G3205(ZAT4)
GH_A05G3181
GH_D03G0327(SCAP1)
GH_D05G3205(CRF8)
GH_D12G0985(EIF4G)
GH_D12G0953(HAT3)
GH_D12G0956(ARABIDILLO-1)
GH_A08G0545
GH_D05G3204
GH_D13G1352
GH_D13G1341(WIP1)
GH_A11G2270
GH_D12G0983
GH_A08G0515(ATL4)
GH_A11G2266
GH_D03G0351(DOT3)
GH_A05G3182
GH_A11G2240(CDC5)
GH_A11G2261
GH_A08G0520(ARAC9)
GH_A11G2241(IAA16)
GH_D03G0311(PCK1)
GH_D03G0337(NET2D)
GH_A05G3200(EDL2)
GH_A08G0542
GH_D13G1328(RGLG4)
GH_A08G0543(AHG11)
GH_D05G3199(ICS2)
GH_D03G0328
GH_A11G2264(MYOB1)
GH_D02G1487
GH_D02G1501(PSBH)
GH_D03G0307(SS5)
GH_D05G3200(DG238)
GH_A05G3218(RAP2.4)
GH_A05G3176
GH_D02G1490
GH_D03G0340
GH_D13G1337(NANA)
GH_A08G0518
GH_A08G0532
GH_D12G0937(SPX3)
GH_A11G2263(FLK)
GH_D12G0970
GH_A05G3192(ATDEK36)
GH_A11G2242(ATAUX2-11)
GH_D03G0338(CCI2)
GH_D02G1493(WRKY75)
GH_D13G1339
GH_A08G0534(DAD1)
GH_D05G3180(ASML2)
GH_A08G0517
GH_D12G0966
GH_A11G2249(PSBW)

Figure S1. Heatmap of the expression level of candidate genes in the ovule and fiber tissues of TM-1. (**A**) The expression level of 80 genes in the ovule tissue; (**B**) The expression level of 86 genes in the fiber tissue. Red indicates high expression, and green indicates low expression.
